# Influence of antipsychotic drugs on microglia-mediated neuroinflammation in schizophrenia: perspectives in an astrocyte–microglia co-culture model

**DOI:** 10.3389/fpsyt.2025.1522128

**Published:** 2025-03-18

**Authors:** Timo Jendrik Faustmann, Franco Corvace, Pedro M. Faustmann, Fatme Seval Ismail

**Affiliations:** ^1^ Department of Psychiatry and Psychotherapy, Medical Faculty, Heinrich Heine University, Düsseldorf, Germany; ^2^ Department of Neuroanatomy and Molecular Brain Research, Medical Faculty, Ruhr University Bochum, Bochum, Germany; ^3^ Department of Neurology, Klinikum Vest, Academic Teaching Hospital of the Ruhr University Bochum, Recklinghausen, Germany

**Keywords:** antipsychotic drugs, glia, neuroinflammation, astrocyte-microglia co-culture model, psychotic disorders, schizophrenia

## Abstract

Schizophrenia is a severe mental disorder with a strong lifetime impact on patients’ health and wellbeing. Usually, symptomatic treatment includes typical or atypical antipsychotics. Study findings show an involvement of low-grade inflammation (blood, brain parenchyma, and cerebrospinal fluid) in schizophrenia. Moreover, experimental and neuropathological evidence suggests that reactive microglia, which are the main resident immune cells of the central nervous system (CNS), have a negative impact on the differentiation and function of oligodendrocytes, glial progenitor cells, and astrocytes, which results in the disruption of neuronal networks and dysregulated synaptic transmission, contributing to the pathophysiology of schizophrenia. Here, the role of microglial cells related to neuroinflammation in schizophrenia was discussed to be essential. This review aims to summarize the evidence for the influence of antipsychotics on microglial inflammatory mechanisms in schizophrenia. Furthermore, we propose an established astrocyte–microglia co-culture model for testing regulatory mechanisms and examining the effects of antipsychotics on glia-mediated neuroinflammation. This could lead to a better understanding of how typical and atypical antipsychotics can be used to address positive and negative symptoms in schizophrenia and comorbidities like inflammatory diseases or the status of low-grade inflammation.

## 1 Introduction

### 1.1 Pathophysiology of schizophrenia

**Figure 1:**
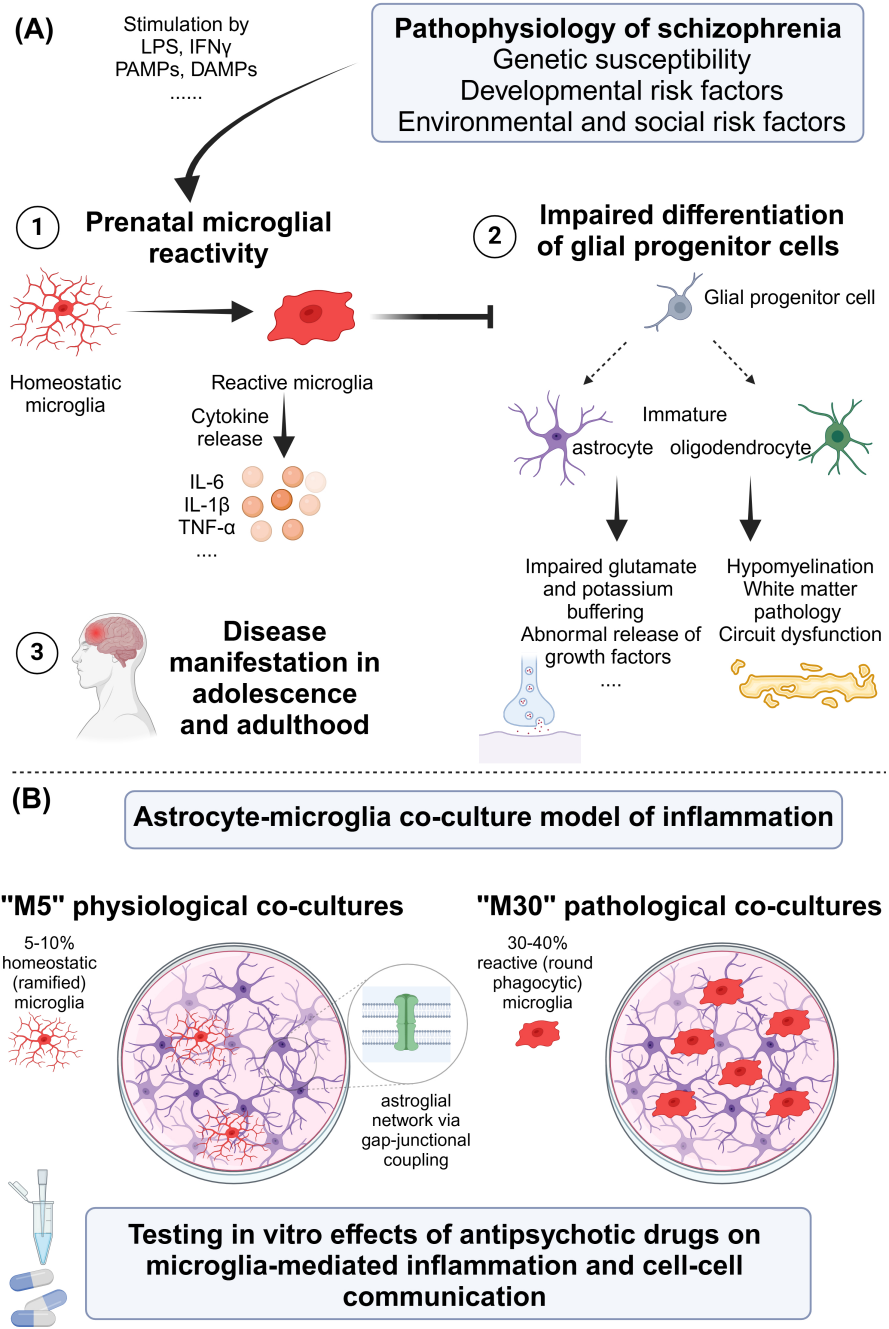
**(A)** Pathophysiology of schizophrenia with focus on microglia. **(B)** Proposal for an *in vitro* astrocyte–microglia co-culture model of inflammation for studying the effects of antipsychotic drugs. DAMP, damage-associated molecular pattern; IFN, interferon; IL, interleukin; LPS, lipopolysaccharide; PAMP, pathogen-associated molecular pattern; TNF, tumor necrosis factor. Created in BioRender. Ismail, F. (2024) https://BioRender.com/z92a369.

Schizophrenia is a severe mental disorder associated with increased mortality and significant morbidity (1). The symptoms of schizophrenia can be divided into positive symptoms (e.g., hallucinations and delusions), negative symptoms (e.g., social withdrawal and anhedonia), mood deficits (e.g., depressed/irritated mood), cognitive deficits (e.g., attention and memory deficits), and motor symptoms (1–3). The pathophysiology of schizophrenia is multifactorial. Genome-wide association studies have identified numerous genetic variants associated with an increased risk of schizophrenia (e.g., DISC1, NRG1, and DTNBP1) by influencing synaptic functions, neurotransmission, and neuronal development and plasticity (4–8). Furthermore, biochemical dysregulation has been discussed considering “the dopamine hypothesis of schizophrenia,” which postulates that overactivity of the mesolimbic dopamine system results in positive symptoms of schizophrenia, while underactivity of the mesocortical dopamine system causes the negative symptoms (9). Another hypothesis postulates the hypofunction of the *N*-methyl-d-aspartate (NMDA) receptor, a subset of the glutamate receptor (10–13). This hypothesis is underlined by NMDA receptor antagonists such as ketamine and phencyclidine (PCP), which can induce symptoms that resemble those of schizophrenia (14–20). Importantly, the inflammatory mechanisms and status of low-grade inflammation have been discussed including the terms “mild encephalitis” (21) and “autoimmune psychosis” (22), the role of prenatal infections (23) and infections during early childhood (24), and the role of peripheral (25) and cytokines in cerebrospinal fluid (CSF) (26, 27). Furthermore, the interrelation between impaired social interactions and inflammatory reactions in schizophrenia has been recognized (28). Moreover, the role of brain microvascular endothelial cells and blood–brain barrier (BBB) dysfunction has been found relevant in inflammation in schizophrenia (29). Structural and functional abnormalities in the brains of patients, which are often present before the onset of clinical symptoms (30, 31), and chronic stress and traumatic experiences have been further discussed (32–34) (**Figure 1A**).

### 1.2 Glial dysfunction in schizophrenia

Glial cells (astrocytes, microglia, and oligodendrocytes) perform a variety of supportive functions for neurons (35). In schizophrenia, various dysfunctions in glial cells have been identified (36, 37).

Astrocytes, the most abundant glial cells in the central nervous system (CNS), have numerous essential roles (e.g., supporting ion and transmitter homeostasis). Findings indicate that astrocytes in schizophrenia exhibit dysregulated glutamate homeostasis. Normally, astrocytes uptake glutamate from the synaptic cleft to prevent neurotoxicity, but in schizophrenia, reduced expression of the glutamate transporters excitatory amino acid transporter 1 (EAAT1) and excitatory amino acid transporter 2 (EAAT2) has been observed, leading to elevated extracellular glutamate concentrations and neuronal hyperexcitability (38–40). Furthermore, in schizophrenia, there is evidence of a dysregulated astrocyte-dependent release of d-serine, a co-agonist for NMDA receptors (41–43). Oligodendrocytes, responsible for the myelination of neurons, also exhibit dysfunctions in schizophrenia. Reduced myelin-associated gene expression and impaired myelination have been observed in the white matter of patients with schizophrenia, which can lead to deficits in neuronal connectivity and signal transmission. These myelination-related deficits have been suggested to contribute to cognitive impairment in schizophrenia (44–48). Additionally, microglial cells have been found to influence oligodendrocytes and their progenitor cells, and as a result, myelination in schizophrenia (49, 50).

#### 1.2.1 The role of microglia in schizophrenia

Microglia are the resident immune cells of the CNS, maintaining neuronal homeostasis, defending against pathogens, and repairing tissue damage. They are highly dynamic cells that adapt their morphology and function to changes in the neuronal environment (51, 52).

Postmortem studies have demonstrated an increase in different inflammatory markers related to microglia in schizophrenia, e.g., an increase in microglial density in cortical gray matter using ionized calcium-binding adaptor molecule-1 (Iba1) (53). Furthermore, an increase in the density of cells [using staining for major histocompatibility complex class II (MHC-II)] morphologically resembling microglia and a change in interleukin-1β (IL-1β), interleukin-6 (IL-6), and interleukin-8 (IL-8) have been found (54). Additionally, the role of an increase in the human leukocyte antigen–DR isotype-positive (HLA-DR+) microglia in the frontal cortex and hippocampus of patients with schizophrenia (55, 56) and calprotectin co-expressed with the microglial marker cluster of differentiation 68 (CD68) has been discussed (57). Other studies have found a change in microglial phenotype rather than a change in the density of cells according to a meta-analysis (58). Twin studies using human induced pluripotent stem cell (iPSC)-derived microglia found an increased expression of inflammatory genes in microglia like MHC-II but no signs of hyperactivation of microglia (59). Furthermore, translocator protein (TSPO) positron emission tomography (PET) and the second-generation radioligand [(11)C]PBR28 *in vivo* confirmed microglial reactivity in patients with schizophrenia and subclinical symptoms (60). However, importantly, postmortem studies did not conclude microglial activation for all the cases, and further *in vivo* studies did not find microglial activation using PET and TSPO in patients with schizophrenia. It can be concluded that postmortem and *in vivo* findings are discussed to be related to brain regions and stage of the disorder and if a antipsychotic treatment was used or not at the same time (36, 37, 61–66). The described state of chronic neuroinflammation could contribute to the pathophysiology of schizophrenia, as inflammatory mediators can impair synaptic transmission and plasticity, resulting in symptoms of schizophrenia (37, 67–69). Furthermore, physiological microglia can perform synaptic pruning, which is crucial for maintaining healthy neuronal circuits. However, the “synaptic hypothesis of schizophrenia” describes a lower synaptic density, which was demonstrated in patients with schizophrenia. Here, genetic and environmental stressors could lead to a microglia-mediated and complement-dependent (among others complement protein C4) elimination of synaptic structures (70–74). Interestingly, microgliosis in patients with schizophrenia, those with depression, and matched controls who committed suicide was found in the dorsolateral prefrontal cortex (DLPC), anterior cingulate cortex (ACC), and mediodorsal thalamus (MD), pointing toward possible further connections between inflammation and psychopathology (75, 76). A further finding that could contribute to the pathophysiology of psychiatric diseases and schizophrenia is the interesting role of microglial priming after early-life infections (first hit) and consequently long-term changes, e.g., on memory after a “second hit” later in life (77, 78). These findings consider the impaired synaptic plasticity in schizophrenia (71). In addition to *in vivo*, postmortem, and animal studies, future directions point toward iPSC models in schizophrenia investigating microglia and neuron interactions (for review, see (65)).

In summary, dysfunctional microglia were found to contribute to the pathophysiology of schizophrenia by promoting inflammatory reactions, abnormal phagocytosis, and reactions to early life stressors. These findings offer potential targets for therapeutic interventions aimed at modulating microglial function (37, 54, 67) (**Figure 1A**). Interestingly, a pharmacological approach (antipsychotics) may mitigate these effects by suppressing microglial activation, offering a dual therapeutic mechanism beyond dopamine modulation (79). Cognitive deficits in schizophrenia are closely tied to microglial overactivation, which exacerbates neurotoxicity through pro-inflammatory cytokines and free radicals. Targeting microglial activation presents a promising avenue for ameliorating cognitive symptoms (80). Also, stress-induced alterations in microglial function have been shown to impact fear memory and extinction deficits, mechanisms that may overlap with schizophrenia pathology. Research has highlighted that aberrant microglial cytokine production affects fear generalization and inhibitory processes in memory, as observed in post-traumatic stress disorder (PTSD) models, and has underscored the necessity for translational studies bridging the gap between animal models and human conditions (81).

## 2 Pharmacological approaches to schizophrenia

### 2.1 Antipsychotics

Antipsychotics are the main therapeutic strategy in schizophrenia and other disorders with psychotic symptoms and can be divided into first-generation (typical) and second-generation (atypical) antipsychotics (82–84). The most valuable difference is the reduced ability to cause extrapyramidal side effects and tardive dyskinesia when using atypical antipsychotics compared to typical antipsychotics. Typical antipsychotics primarily provide modulations of dopaminergic transmission compared to a more serotonergic transmission in cases of atypical antipsychotics. The most prominent antipsychotics are haloperidol as a typical antipsychotic and clozapine as an atypical antipsychotic, which is also useful in cases of treatment-resistant schizophrenia. Different effects on positive and negative symptoms in schizophrenia when using antipsychotics have been discussed. Clozapine has a strong effect on positive symptoms like hallucinations and delusions with an improvement in social functioning. Haloperidol was found to have an effect on positive [scale for the assessment of positive symptoms (SAPS)] and negative symptoms [scale for the assessment of negative symptoms (SANS)], and negative symptoms were found to be independent of positive ones (85–88).

### 2.2 Effects of antipsychotics on microglia and microglia-mediated neuroinflammation

#### 2.2.1 Clinical evidence (effects on microglia and cytokines)

Increased activity of microglia and pro-inflammatory cytokines was found to be important in the pathophysiology of schizophrenia. These inflammatory findings were potentially influenced by antipsychotics (89, 90). A cytokine imbalance in serum was found to be an important biomarker in treatment-resistant schizophrenia, and concentrations vary between acute and chronic stages (25, 91). In a meta-analysis, antipsychotic treatment was found to reduce cytokine levels in patients with schizophrenia *in vivo* and here, especially IL-6 levels (92). In contrast, using PET studies for TSPO as a marker for microglial activity revealed a significant increase in the marker in schizophrenia patients with antipsychotic treatment. Furthermore, the marker correlated with negative symptoms using the Positive and Negative Syndrome Scale (PANSS) (66, 93). Other data found a decrease in TSPO in antipsychotic-treated patients (94). Moreover, an increase in TSPO binding in rats upon clozapine treatment was found (95). Reviewing clinical data, no clear conclusion could be drawn about antipsychotic drugs influencing microglial cells (96). Nevertheless, microglial proliferation and morphological changes resembling microglial activation were found *in vivo* in rats during an 8-week treatment with haloperidol and olanzapine (97).

#### 2.2.2 Experimental evidence

##### 2.2.2.1 Typical antipsychotics

Haloperidol is a typical and strong dopamine D2 receptor blocker (98). It reduces pro-inflammatory action in C57/BL6 murine microglial cells (BV-2 microglia) (99) and increases brain-derived neurotrophic factor (BDNF), transforming growth factor-β (TGF-β), and neurotrophin-3 (NT-3) gene expression in microglial cells from newborn Wistar rats (100). Interestingly, haloperidol did not change microglial density in an *in vivo* rat model (101) and did not prevent microglial activation in a PCP model of psychosis in rats (102). Furthermore, a high-fat diet increased microglial expression in rats, and this was not found in a combination of diet and haloperidol (103). Expression of OX-42 protein and IL-6 expression was decreased, and extracellular signal-regulated kinase (ERK) and signal transducer and activator of transcription 3 (STAT3) was suppressed by haloperidol in lipopolysaccharide (LPS)-activated microglia (104). Microglial proton currents in BV-2 microglial cells were inhibited by haloperidol and could contribute to the anti-inflammatory effects of antipsychotics on microglia.

Furthermore, the same was found for chlorpromazine, another typical antipsychotic (105). Additionally, chlorpromazine reduced secretion of interleukin-2 (IL-2) and IL-1β in mixed glial cultures (106) and acts as a microglia Kv1.3 (voltage-gated potassium channel) channel inhibitor (107).

Flupentixol decreased IL-2 and IL-1β release by microglial cells, and trifluperidol reduced IL-1β and IL-2 release by mixed glial cultures (108). Both antipsychotics also reduced the nitric oxide (NO) and tumor necrosis factor-alpha (TNF-α) release from LPS-influenced microglial cultures (109).

Spiperone attenuates TNF-α production, expression of IL-1β and TNF-α, and nuclear translocation of the p65 subunit of nuclear factor kappa B (NF-κB) in BV-2 microglia and reduces microglia-mediated cell death in microglia/neuron co-cultures (110).

##### 2.2.2.2 Atypical antipsychotics

Clozapine is an atypical antipsychotic with a strong influence on serotonergic transmission [blockade of 5-HT(2A) receptors] and reduced blockade of dopamine D2 receptors in the ventral and dorsal striatum (111). In a mouse model of experimental autoimmune encephalomyelitis (EAE), clozapine regulated the iron-impaired microglial function and reduced the release of IL-6 and neuronal phagocytosis (112). Furthermore, it reduced the inflammatory NOD-, LRR-, and pyrin domain-containing protein 3 (NLRP3) pathway in a polyriboinosinic–polyribocytidylic acid (poly(I:C))-stimulated primary microglial cell culture model (113). Clozapine reduces the inhibition of calcium/calmodulin/Akt-mediated NF-κB activation in microglia (114), and clozapine-induced neuronal protection was microglial mediated in an LPS-induced model of inflammatory neurodegeneration using neuron–glia cultures (115). Interestingly, clozapine can reduce proton currents in BV-2 microglial cells, which could be considered as an anti-inflammatory effect (116).

Aripiprazole is a partial agonist at the dopamine D2 and serotonin 5-HT(1A) receptor and an antagonist at the serotonin 5-HT(2A) receptor (117). Aripiprazole inhibited inflammatory mechanisms in a poly(I:C)-induced microglial activation model in mice (118). An interferon-gamma (IFN-γ)-induced microglial activation was found to be attenuated by aripiprazole via intracellular calcium regulation *in vitro* (119). In BV-2 microglial cells, aripiprazole reduced the pro-inflammatory action and expression of anti-inflammatory markers (99). Interestingly, aripiprazole and minocycline inhibited damage of oligodendrocytes via the inhibition of IFN-γ-activated microglia (50).

Quetiapine inhibited NO generation and TNF-α release from activated microglia (120). In a transgenic mouse model of Alzheimer’s disease, it decreased β-amyloid-(1-42) (Aβ(1-42))-induced activation of primary microglia by attenuating pro-inflammatory cytokines and in primary microglia stimulated by Aβ(1-42) via activation of the NF-κB pathway (121). Furthermore, quetiapine inhibits microglial activation via neutralization of abnormal intercellular calcium homeostasis in a cuprizone mouse model (122).

Risperidone reduced the pro-inflammatory activation of BV-2 microglial cells (99) and deactivated IFN-γ-induced microglia (123). Moreover, risperidone reduced the expression of OX-42 protein and decreased the IL-6 and TNF-α production via STAT3 in LPS-activated microglia (104).

Olanzapine reduced NO release in an LPS-activated mouse microglia cell line N9 (124). A high-fat diet increased microglial expression in rats, and this was not found in a combination of diet and olanzapine (103).

In summary, mitogen-activated protein kinase (MAPK) was found to be important in the activation of BV2 microglia by LPS and ERK in IFN-γ activated BV2 microglia (125, 126). As previously described, antipsychotics seem to modulate intracellular signals like MAPK, calcium homeostasis, NF-κB, and protein kinase C (PKC), which further inhibit nuclear activation and cytokine production and release by microglia (89). These discussions could contribute to the described decrease in pro-inflammatory cytokines when using antipsychotics. Ca^2+^ is a main point of interest, as it is an activator of PKC and found to be dysregulated in schizophrenia (127). It could also be influenced by aripiprazole, for example (89).

Taken together, psychopathologies in psychiatric diseases have been known to be associated with the sensitization of glial and particularly microglial cells, which could be influenced by psychotropic drugs like antipsychotics, contributing to the microglia hypothesis of schizophrenia. Here, the involvement of microglia and oligodendrocytes in negative and cognitive symptoms in schizophrenia was discussed (37, 46, 48, 66, 128, 129). As described above, the known antipsychotics (typical and atypical) can contribute to a possible anti-inflammatory effect concerning microglial cells (**Table 1**). Furthermore, in this vein, specific effects on positive and negative symptoms of schizophrenia when using antipsychotic drugs were discussed. In contrast, microglial reactivity could not be concluded as ubiquitous in schizophrenia and psychiatric diseases concerning postmortem and *in vivo* studies in humans. Postmortem microglial markers were found to be increased or unchanged, and the reason for this is not clear; similar findings were found to be related to PET studies. The contradictory findings in postmortem studies and PET investigations may be attributed to differences in the sensitivity of the methods capturing different stages of microglial activity (36, 62, 130). Furthermore, the role of antipsychotics in the treatment of psychiatric diseases and their role in glial reactivity were discussed. The interrelations among neurons, astrocytes as part of the tripartite synapse, and microglia underline the complex glial mechanisms influenced by antipsychotics (131). Nevertheless, using antipsychotics could be one way of regulating a microglial immune response.

**Table 1:** Literature on antipsychotic drugs influencing microglia.

Author	Glial culture (*in vitro*/*in vivo*)	Antipsychotic drug	Effect
Lee et al. (107)	Mouse medial prefrontal cortex treated with LPS	Chlorpromazine	Acts as a microglia Kv1.3 channel inhibitor
Long et al. (104)	BV-2 microglia activated with LPS and treated with minocycline	Haloperidol, risperidone	Strong anti-inflammatory effect of risperidone (IL-6 and TNF-α) and minocycline (IL-6, TNF-α, and IL-1β) via MAPK and JAK-STAT
Conen et al. (93)	*In vivo* imaging of microglia activity (TSPO)	Antipsychotics	TSPO binding potential was higher in patients with schizophrenia and antipsychotic medication
Ceylan et al. (112)	Iron-impaired microglia in EAE mouse model	Clozapine	Reduces release of IL-6 and normalization of neuronal phagocytosis
Racki et al. (99)	BV-2 microglia	Haloperidol, risperidone, aripiprazole	All reduce pro-inflammatory action; mTORC1 activity reduces with aripiprazole
Maredia et al. (103)	Microglia of male Sprague Dawley rats in *in vitro* autoradiography and high-fat diet	Haloperidol, olanzapine	High-fat diet but not antipsychotics increases activated microglial expression
Giridharan et al. (113)	Unstimulated and poly(I:C)-stimulated primary microglial cell cultures	Clozapine, risperidone, haloperidol	Clozapine exhibits anti-inflammatory effects via the NLRP3 pathway
Namjoo et al. (100)	Cultured rat microglia	Haloperidol	Increases gene expression (TGF-β, BDNF, and NT-3)
Jeon et al. (114)	Microglial cells LPS activated	Clozapine	Clozapine has an anti-inflammatory effect via inhibition of calcium/calmodulin/Akt-mediated NF-κB activation
Bloomfield et al. (101)	Microglia of male Sprague Dawley rats—TSPO expression	Haloperidol	Microglia cell density, morphology, and TSPO expression unchanged
Di Biase et al. (94)	*In vivo* imaging of microglia activity (TSPO) in schizophrenia	Antipsychotics	TSPO was decreased in medicated patients
Holmes et al. (66)	*In vivo* imaging of microglia activity (TSPO) in schizophrenia	Antipsychotics	TSPO was elevated in medicated patients
Sato-Kasai et al. (118)	Murine and human microglial cells (poly(I:C)-stimulated)	Aripiprazole	Inhibition of TNF-α via MAPK—possibly via Ca^2+^ and TRPM7
Wang et al. (122)	Microglia in cuprizone-induced demyelination mouse model; microglial N9-cells activated with LPS	Quetiapine	Reduces recruitment and activation of microglia/macrophage; inhibits NO and TNF-α release; reduces translocation of NF-κB p65 subunit; and reduces LPS-induced and STIM1-mediated intercellular calcium homeostasis
Cotel et al. (97)	Microglia of rats *in vivo*	Haloperidol, olanzapine	Proliferation and activation of microglia in the naïve rat brain
Shin et al. (116)	BV-2 microglia	Clozapine, olanzapine, risperidone	Clozapine and olanzapine reduce proton currents (clozapine even at therapeutic doses)
Zhu et al. (121)	Glial activation in mice (APP/PS1 mice—*in vitro* and *in vivo*) and primary microglia	Quetiapine	Attenuates glial activation, reduces pro-inflammatory microglia, and inhibits NF-κB pathway
Shin et al. (105)	BV-2 microglia	Chlorpromazine, haloperidol	Inhibit voltage-gated proton currents
Hu et al. (115)	Primary cortical and mesencephalic neuron–glia cultures, primary neuron-enriched and microglia-enriched cultures, and HAPI microglial cell line activated with LPS	Clozapine	Neurotoxicity was reduced, and ROS production and TNF-α were reduced
Zheng et al. (110)	BV-2 microglia and microglia/neuron co-cultures	Spiperone	Attenuates TNF-α production and expression of IL-1β and TNF-α, nuclear translocation of the p65 subunit of NF-κB
Bian et al. (120)	Activated microglia	Perospirone, ziprasidone, quetiapine	All inhibit NO generation, and perospirone and quetiapine inhibit TNF-α release
Kato et al. (119)	Murine microglial cells—IFN-γ activated	Aripiprazole	Inhibition of NO and TNF-α generation may be via suppression of intracellular Ca^2+^
Danovich et al. (95)	C6 rat glioma cells, MA-10 mouse Leydig tumor cells, male Sprague Dawley rats (TSPO binding)	Clozapine, risperidone, thioridazine, sulpiride	Clozapine increases TSPO binding in both cell lines and in rats
Kato et al. (123)	Microglial cells—IFN-γ activated	Risperidone, haloperidol	Risperidone reduces NO, iNOS, TNF-α, IL-6, and IL-1β
Hou et al. (124)	Mouse microglial cell line N9 activated with LPS	Clozapine, olanzapine, haloperidol	Olanzapine significantly inhibits NO release
Labuzek et al. (106)	Rat mixed glial and microglial cell cultures activated with LPS	Chlorpromazine, loxapine	Reduce IL-1β and IL-2 from both cultures
Kowalski et al. (108)	Rat mixed glial and microglial cell cultures	Flupentixol, trifluperidol	Reduce IL-1β and IL-2 from both cultures
Kowalski et al. (109)	Rat microglial cell cultures activated with LPS	Flupentixol, trifluperidol	Reduce TNF-α and NO
Nakki et al. (102)	Microglia of 30- to 90-day-old rats and exposure to ketamine and PCP	Haloperidol	Failed to prevent microglial activation

BDNF, brain-derived neurotrophic factor; BV-2 microglia, C57/BL6 murine microglial cells; EAE, experimental autoimmune encephalomyelitis; Kv1.3, voltage-gated potassium channel; IL-6, interleukin-6; IL-2, interleukin-2; IL-1β, interleukin-1β; IFN-γ, interferon-gamma; iNOS, nitric oxide synthase; JAK-STAT, Janus kinase and signal transducer and activator of transcription; LPS, lipopolysaccharide; MAPK, mitogen-activated protein kinase; mTORC1, mammalian target of rapamycin complex 1; NO, nitric oxide; NF-κB pathway, nuclear factor kappa B pathway; NLRP3, NOD-, LRR-, and pyrin domain-containing protein 3; NT-3, neurotrophin-3; PCP, phencyclidine; poly(I:C), polyriboinosinic–polyribocytidylic acid-stimulated; ROS, reactive oxygen species; STIM1, stromal interaction molecule 1; TGF-β, transforming growth factor-β; TNF-α, tumor necrosis factor-alpha; TRPM7, transient in receptor potential in melastatin 7; TSPO, translocator protein.

## 3 Proposal for an *in vitro* astrocyte–microglia co-culture model for investigation of antipsychotic drugs

The astrocyte–microglia co-culture model of inflammation was developed in 2003 by Faustmann et al. to investigate the physiological as well as pathological inflammatory conditions in the brain in relation to the percentage and activation state of microglia (132) (**Figure 1B**). The astrocytes and microglia were obtained from the brains of postnatal Wistar rats (postnatal days 0–2) and were prepared according to an established protocol (132, 133).

The physiological astrocyte–microglia co-culture model (so-called M5) contains 5%–10% microglia with the predominantly homeostatic ramified phenotype (formerly known as resting ramified). The pathological, inflammatory astrocyte–microglia co-culture model (so-called M30) contains 30%–40% microglia with predominantly reactive phenotype (formerly known as activated, round phagocytic) (132, 134). Treatment of M5 co-cultures with the pro-inflammatory cytokines TNF-α, IL-1β, IL-6, and IFN-γ led to microglial reactivity, whereas treatment of M30 co-cultures with the main anti-inflammatory cytokine transforming growth factor-β1 (TGF-β1) caused a reduction of microglial reactivity. In addition, treatment of M5 co-cultures with the anti-inflammatory cytokine interferon-beta (IFN-β) prevented the pro-inflammatory effects of TNF-α, IL-1β, and IFN-γ (134). Further, the study findings of our co-culture model revealed a functional relationship between microglial activation states and astroglial coupling (132).

Interestingly, previous findings point to the fact that functional abnormalities of tripartite synapses, contributing to dynamic signaling between pre- and post-synaptic neurons as well as astrocytes, can be involved in the pathophysiology of schizophrenia and associated cognitive impairments (135). The gliotransmitter release through vesicles, hemichannels, and reverse transport of astrocytes plays an important role in tripartite synaptic transmission. Astrocytes are connected with each other and other cell types via connexin (Cx)-based gap junctions, mainly consisting of Cx43 and Cx30 (136). Connexins can also form hemichannels that enable the connection between intra- and extracellular spaces. Study findings show that atypical antipsychotics such as clozapine, brexpiprazole, and quetiapine increased astroglial Cx43 containing hemichannel activities, resulting in enhanced tripartite synaptic glutamatergic transmission (135). *In vivo* chronic treatment of mice with clozapine caused increased Cx43 expression in the prefrontal cortex; however, haloperidol led to a decrease in Cx43 (137).

The inflammatory astrocyte–microglia co-culture model offers the possibility to examine the inflammatory states including microglial morphology and Cx-based gap junctional coupling, which are also involved in the pathophysiology of schizophrenia. Consequently, the co-culture model can have a long-term impact on the treatment of schizophrenia, such as in the development of new drugs. For example, connexin-based channels could serve as a target for new drugs in schizophrenia, modulating the gap junctional coupling, and indirectly, the microglial reactivity. Study findings suggest that astrocytic deficits in the dorsolateral prefrontal cortex can disrupt the neuron–glia interactions, resulting in a dysfunctional effect on prefronto-striatal circuits in schizophrenia (138). Repairing astrocytic dysfunction could offer new therapeutic options for schizophrenia. Our astrocyte–microglia co-culture model has more advantages for studying cellular interactions compared to only astrocyte or microglia monocultures (133). Our co-culture model mimics natural inflammation because of the preparation method, allowing concomitant proliferation of astrocytes and microglia (132). In contrast to this, other astrocyte–microglia co-cultures consist of a mixture of two primary cultures (astrocytes and microglia) cultivated together in different ratios. Of course, there is a limitation of our model to tricultures, including neurons in addition to astrocytes and microglia (139). Nevertheless, our model is robust and suitable for schizophrenia research in the first step before further steps are taken in animal models.

In further studies, the *in vitro* model was already used for the investigation of pharmaceutical effects on glia-mediated neuroinflammation and cellular interactions (133). Different groups of neuropsychiatric drugs including anti-seizure medication and mood-stabilizing drugs/antidepressants were already investigated in the co-culture model (139–147). For example, venlafaxine as well as doxepin and amitriptyline reduced the microglial reactivity, leading to the attenuation of microglia-mediated neuroinflammation (141, 146). Next, the co-culture model can be suitable for testing the possible pro-/anti-inflammatory effects of antipsychotic drugs.

## 4 Conclusion

Psychotic disorders encompass a broad spectrum of psychiatric conditions including schizophrenia as one of the leading psychotic disorders with a strong lifetime impact on patients’ health and wellbeing. In recent years, studies have been conducted to better understand the cellular neurobiology of schizophrenia, particularly with regard to cellular-mediated neuroinflammation (28, 79, 148). The low-level inflammation concept of schizophrenia was linked to risk genes promoting inflammation, prenatal maternal and early childhood infections with microglial reactivity, and an increase in cytokines, environmental stress factors, and alterations of the immune system (69). In addition, autoimmune factors such as anti-NMDA receptor antibodies contributing to psychosis were discussed (22). Experimental and neuropathological evidence suggests that reactive microglia have a negative impact on the differentiation and function of oligodendrocytes, glial progenitor cells, and astrocytes, which results in the disruption of neuronal networks and dysregulated synaptic transmission, contributing to the pathophysiology of schizophrenia (148) (**Figure 1A**). Following this, research focusing on therapeutic approaches modulating microglia-mediated neuroinflammation has potential. However, it is currently even more exciting to consider whether antipsychotics used in everyday clinical practice have anti-inflammatory properties in relation to microglia-mediated neuroinflammation.

### 4.1 Future perspectives

Research focusing on inflammatory disease mechanisms in many neuropsychiatric disorders has recently expanded. Also, various immunotherapeutic approaches have been developed, e.g., immunotherapy for Alzheimer’s disease with monoclonal antibodies removing abnormal β-amyloid (Aβ) from the brain and preventing disease progression (149–153). Preliminary data have been about the effects of monoclonal antibodies acting by directly neutralizing cytokines or by binding cytokine receptors in schizophrenia (154). Subsequently, aiming at the involvement of microglia in schizophrenia, the use of *in vitro* models such as our astrocyte–microglia co-culture model of inflammation can help to better understand the underlying pathomechanism by testing the effect of antipsychotic and anti-inflammatory drugs (**Figure 1B**). This could lead to a better understanding of how typical and atypical antipsychotics can be used to address positive and negative symptoms in schizophrenia and comorbidities like inflammatory diseases or the status of low-grade inflammation.
